# Cyclists' Perception of Maintenance and Operation of Cycling Infrastructure—Results From a Norwegian Survey

**DOI:** 10.3389/fpsyg.2021.696317

**Published:** 2021-07-14

**Authors:** Ole Aasvik, Torkel Bjørnskau

**Affiliations:** Institute of Transport Economics, Oslo, Norway

**Keywords:** operation and maintenance, cyclists, survey, evaluation, infrastructure

## Abstract

The Norwegian authorities want to limit the extent of car use in city areas to existing levels. Such a limitation would help combat climate change, improve health of citizens, and alleviate congestion. This implies that any further increase in transport needs will have to be met by walking, cycling and use of public transport. Reaching this ambitious goal requires knowledge about cyclists' preferences concerning operation and maintenance (M&O) of roads and foot/cycle paths. Previous research suggests that M&O have great implications for travel mode choice, bicycle route/path choice, safety, security, and comfort. With the need to serve bicyclists of all ages and genders, this study additionally explores which M&O of roads and foot/cycle the different demographic groups perceive positively or negatively. This article reports results from a nationwide survey in the summer of 2019. Two thousand three hundred seventy-six cyclists across Norway (55% male; 29% <40; 17% >60) participated to determine the cyclists' perceptions about year-round M&O of roads and foot/cycle paths. Respondents, rather than being randomly selected, completed an internet-linked survey. The variables included maintenance of foot/cycle paths in terms of salt and snow plowing and operation and maintenance of roads in terms of glass, holes/bumps, and conditions. Our results suggest that female cyclists suffer more from adverse conditions than do males. We also find that males are more likely to cycle during winter, which is an additional indication that adverse conditions affect women and men differently. Surprisingly, older cyclists report to be less affected by poor conditions than younger cyclists. Self-selection to participate in the survey among older cyclists might be an important explanation for this result. Cycling conditions vary greatly between geographical areas, reflecting the large climatic variations across Norway. Most respondents have experienced a cycle accident where conditions contributed, and many sometimes forfeit cycling due to adverse conditions. Implications for future research and practice of M&O are discussed.

## Introduction

The Norwegian authorities have stated that they want to limit car use in city areas to existing levels (“nullvekstmålet”). They are in line with a global movement that seek to open city centers up to pedestrians and bicyclists (Wolfe, 2018). This limitation of cars could also help combat climate change and improve health of citizens. This implies that any future growth in transport needs must be met by increased use of public transport, walking, and cycling. Hence it is a political goal to increase the cycling share in city areas across Norway (Espeland and Amundsen, 2012), with some cities, such as Oslo, going even further in suppressing car traffic in urban areas. However, recent figures suggest that most city areas in Norway have <10 percent cycling share (Lunke and Grue, 2018), and despite the fact that both local and central government have made goals of increasing this share, a recent evaluation reveals that the cycling share does not increase in line with the ambitions (Lunke and Grue, 2018).

There are basically two types of designated cycle infrastructure implemented in Norway: separated paths for bicyclists and pedestrians, and bicycle lanes in the ordinary streets. Bicycle lanes, normally with red colored asphalt or paint, are most common in the city centers, whereas bicycle (and pedestrian) paths dominate outside cities. To mix pedestrians and cyclists on the same infrastructure is common, and it is also allowed for cyclists to cycle on foot paths, sidewalks and in pedestrian streets.

Bicycle infrastructure is normally built according to a uniform standard defined by the Norwegian Public Roads Administration, but local adjustments are often applied. Still, in all the studied areas, the bicycle infrastructure is more or less similar, consisting of bicycle lanes in inner city centers and bicycle paths shared with pedestrians outside city centers. New bicycle and pedestrian paths are normally built with pavement for pedestrians, separating them from bicyclists.

In most urban areas in Norway, and in particular in Oslo, there has been a steady and huge increase in the use of bicycle lanes during later years. The Oslo city authorities have ambitious plans to build a city-wide bicycle network and 52 km of new bicycle infrastructure were built from 2016 to 2020. Today Oslo, with a population of 700 000 inhabitants, has a total bicycle network of ca. 250 km (Oslo City Counsil, 2021) and Trondheim has ca. 150 km (Norwegian Public Roads Administration, 2021). For other cities in Norway, there are no good estimates for the size of bicycle networks, but there is reason to believe that it is increasing.

Infrastructure design could be important to achieve a transport modality shift and increase bicycle ridership. Literature reviews have suggested that building accommodations for cyclists and extending the network of cycle paths and lanes increase cycling (Buehler and Dill, 2016). Others found that, among other factors, separation and security was important in increasing the cycling share (Hesjevoll and Ingebrigtsen, 2016). Continuous, separate cycling infrastructure seems to be an essential part of creating a safe and encouraging cycling environment. This seems particularly true for inexperienced cyclists who may feel insecure, and who must be encouraged to cycle if one wants to increase the cycling share. While important, this paper will focus on the maintenance and operation (M&O) of surfaces wherever cyclists ride. No matter how important the infrastructure is, it would need to be properly maintained and operated to have the best possible impact (Hesjevoll and Ingebrigtsen, 2016).

Increasing the share of transport done by cycling could increase the number of traffic injuries (Bjørnskau, 2018). If relatively more people walk or cycle one would expect particularly light injuries to increase. This could attenuate the shift toward active mobility. It is suggested that improved M&O could mitigate such an effect and facilitate the shift safely (Bjørnskau, 2018). Operation includes measures that keep roads functioning well in daily use, such as snow removal and measures to improve road grip during winter. Maintenance means taking care of the physical infrastructure in a longer perspective, such as maintaining the standard of road surfaces with quality requirements. Road conditions have been pointed to as a significant factor for many accidents, especially among inexperienced cyclists (Janstrup et al., 2018). While it is suggested that improved M&O would be an integral part of this shift, we do not yet know much about what kind of improvement would be most efficacious.

Demographic factors have been suggested as determining the influence of M&O on cyclists (Svorstøl et al., 2017). Women seem to be more impacted by poor M&O than men. This is in line with other research finding that gender plays a role in bicycle use and the impact of cycling related psychological distress associated with cycling (Useche et al., 2018b). Differences in perception could translate to differences in behavior, and help explain why males tend to use bicycles more often, and take greater risks, than women (Sahlqvist and Heesch, 2012; Useche et al., 2018a). Crash rates have been found to be aptly explained through execution of risky behavior and risk perception (Useche et al., 2019). Behavioral factors have also been documented to interact with environmental hazards in leading to accidents (Billot-Grasset et al., 2016). While these demographic differences in risk perception and behavior are well-documented, little is known about how to mitigate them through M&O.

Norwegian roads and infrastructure face seasonally dependent challenges, and winter-related conditions impact elderly and physically disabled individuals more severely. The consequences of suboptimal M&O are far reaching, and influence route choice, choice of transportation mode, whether to travel, and both perceived and actual safety. Thus, proper and improved M&O could greatly boost security and accessibility of cycling and help reach the ambitious goals of increasing the share of cyclists to 20% in Norwegian city areas. Research has suggested that improved winter maintenance could increase bicycle use by 18% and reduce reliance on cars (Bergström, 2002). Other research has also pointed out gender differences in the use of cycling infrastructure, and suggests more research needs to be done to investigate effective strategies for recruiting female cyclists (Garrard, 2003).

While infrastructure is necessary for a safe and pleasant trip by bike, proper and prioritized M&O is also a prerequisite for making cycling more attractive and available to more people (Garrard, 2003; Karhula, 2014). The mutual interdependence has been noted by recent investigations (Ekblad et al., 2016). Therefore, it is critical that we investigate how to best prioritize M&O for different population groups, and to tackle the most important factors first (Svorstøl et al., 2017). Mode choice has been found to differ between seasons in northern countries (Bergström, 2002), which further emphasizes the need for improved M&O.

A recent survey investigated cyclists' perceptions of M&O across Norway (Johansson and Bjørnskau, 2020). Results suggest that many different conditions have impacts on the comfort and ease of travel, as well as whether one chooses to cycle or use other modes of transport. In regular summer conditions potholes and unevenness, sand, gravel and shards of glass are reported as most problematic. Many report poor snow plowing on cycle paths during winter. More debate surrounds the use of road salt. While it helps keeping roads bare, it deteriorates bicycles and asphalt. Salt use varies across Norway. The survey also reiterated the finding that different demographics react differently to poor conditions; women over 60 years seem particularly troubled by it. The survey report, while uncovering cyclists' perceptions, does not employ any multivariate analyses to investigate the relationships between the variables to a greater extent (Johansson and Bjørnskau, 2020).

### Research Questions

Previous research has suggested that M&O is important to increase the cycling shares and to do so safely. It has been suggested that adverse conditions affect different demographic differently. Additionally, Norway is a diverse geographical country with distinct issues in summer and winter. In this paper, we will investigate demographic differences regarding M&O as well as considering geographical effects. In light of previous research and experiences, we have formulated the following hypotheses:

1) Female cyclists will be more affected by poor cycling conditions than men
2) Older participants will be more affected by poor cycling conditions than younger participants
3) Maintenance and operation affect whether one chooses to cycle
4) Conditions both in summer and winter impact different groups differently

We will also investigate other factors regarding demographic and geographic differences in how people experience M&O.

## Methods

### Study Design and Sample

This paper presents results from a survey with a cross-sectional design administered during June, July and the start of August 2019. Recruitment for this study was done in multiple ways. We wanted a broad geographical representation of Norwegian cyclists. Six different recruitment strategies were used. (1) We sent out invitational emails to people who had participated in previous surveys and agreed to be contacted for future surveys. (2) The Norwegian Cyclists' Association (SLF) included a link to the survey in their newsletter. (3) We had a paid advertisement on Facebook. (4) Employees in the city of Oslo also received an invitational email. (5) Several bicycle repair shops agreed to have posters about the study put up. (6) Project partners in the Norwegian Public Roads Administration shared the link to the survey. Additionally, some people reported being recruited through other channels, such as word of mouth. At every instance, it was made clear that we wanted participants who cycled.

We ended up with a total sample of 2,556 cyclists who represented the largest urban areas across Norway. Due to the many channels of recruitment and low control of the spread of the invitation, it is impossible to calculate any response percentage (see Table 1 for population data). Of the total 2,556 respondents who completed the survey, 2,376 (93%) reported that they regularly used a cycle at the time of the survey. Only these 2,376 were included in the analyses.

**Table 1:** Independent variables in the analyses; all except “gender” and “often cycled” are categorical.

**Independent variables**	**%**	**N**	**Population (2019)**
**Area**	100	2,376	
Other	14	338	2,987,606
Akershus	7	176	624,055
Bergen	8	189	283,929
Buskerud	6	137	283,148
Jæren	13	312	201,801
Oslo	16	386	681,067
Trondheim	19	450	198,219
Tromsø	16	388	76,975
**Gender**	100	2,376	
Male	55	1,308	
Female	45	1,068	
**Age groups**	100	2,376	
<40	29	699	
40–49	29	687	
50–59	24	578	
>60	17	411	
**Bike type**	100	2,376	
Other	16	369	
Hybrid	28	665	
MTB	14	341	
Racer	15	364	
E-bike	27	637	
**Often cycled**	100	2,376	
<4 days per week	38	908	
More than 4 days per week	62	1,468	

### Questionnaire

Participants were informed at multiple points that the questionnaire was going to survey them about their thoughts and actions as cyclists regarding M&O. All included variables are presented in Tables 1, 2. Included variables do not measure absolute values but are relative in nature. Many of the included variables are highly skewed. To alleviate this problem, several variables have been dichotomized. This is not always advisable, but problems associated with skewness can be addressed with such an approach (MacCallum et al., 2002). For some variables, not even the dichotomization assures an even split.

**Table 2:** Dependent variables included in multiple different analyses.

**Dependent variables**	**%**	**N**
Accident where road conditions contributed	100	711
At least one = 1	74	525
Authorities use too much salt on foot and cycle paths	100	1,418
Agree = 1	44	620
How satisfied are you with snow plowing on foot and cycle paths	100	1,586
Well satisfied = 1	47	740
How problematic are shards of glass on the road	100	2,331
Problematic = 1	49	1,136
How problematic are holes and bumps on the road	100	2,369
Problematic = 1	51	1,212
Do you sometimes forfeit cycling during summer due to poor road conditions?	100	2,376
Yes = 1	30	720
Winter Cycling	100	2,376
Never/sometimes = 0	55	1,309
Often = 1	45	1,067

*All variables are dichotomous. Percent and number of respondents given value 1 on each variable is reported*.

Variables included were developed on the basis of literature review, stakeholder's comments and previous interviews (Johansson and Bjørnskau, 2020; Rynning et al., 2020). The questionnaire was designed to explore these factors and not to be exhaustive.

#### Independent Variables

Table 1 provides an overview of the independent variables included in the analyses.

**Figure 1:**
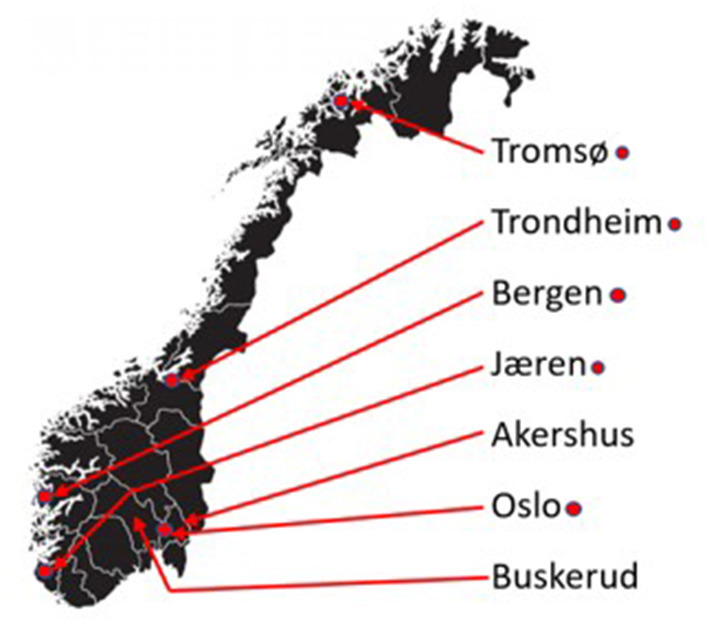
A map of Norway highlighting included areas.

Geographical area of the participant was determined using the postal code they reported. They entered this code, and the code was translated to area using an official table. The areas were then divided based on participant numbers and topographical similarity. The total population of Norway in 2019 was 5,356,800. *Akershus* is a region surrounding the capital (Oslo), and it is mostly a car-based suburb, normally with clear seasonal variations with ice and snow in the wintertime. *Buskerud* is a more sparsely populated rural region with even larger seasonal variations. *Jæren* includes Stavanger city and surrounding areas and is, like *Bergen*, characterized by an oceanic climate with minimal snow. Both *Bergen* and *Jæren*, due to their climate, focus more on combating ice than plowing snow. Both *Trondheim* and *Tromsø* have relatively stable oceanic climates, but experience snow during winter months, in particular Tromsø, with snow depths frequently over 1 m in winter. *Oslo* is the capital in Norway, a densely populated city area situated on the coast with seasonal climactic variations, also experiencing snow and ice in winter. The rest of the reported postal codes were coded as *Other* and includes areas from across Norway. The population figure for *Other* given in Table 1 is the sum of the reported regions subtracted from the total population of Norway, see Figure 1 for an overview of included areas.

Participants' age group was recoded from an original 10-year interval as presented in the questionnaire. They were recoded as “ <*40*,” “*40–49*,” “*50–59*,” and “>*60*” to create more even cell sizes while maintaining groups of younger, middle-aged, and older participants. The original interval consisted of 10 participants under 20 years and only five participants older than 80.

Some cyclists use several bicycles. Participants were asked “*What kind of bike/bikes do you use?*” Those who reported using multiple types of bikes were recoded to one single bike type. They were assigned to the bike type with fewest respondents; most participants with multiple bikes were assigned to either “*E-bikes*” or “*racer*” as these were underrepresented. These respondents have experiences using multiple bikes, and will increase the robustness of results for categories with less respondents. In addition to these two types, we also included “*Hybrid*” bikes and “*MTB*.” Hybrid bikes are a compromise between terrain and classic bikes, while mountain bikes (MTBs) are more terrain-oriented i.e., with good suspension. The rest of the respondents were grouped as using “*Other*” bikes. Because the survey was administered during summer, some who cycle during winter may use a different type of bike for that season.

Cycling frequency or how often participants cycle was measured asking “*How often do you normally cycle this time of year?*” Participants had six alternatives ranging from “1–*Never*” to “6–*More than 4 days per week.”* Those who never cycled (category 1) were excluded from analyses. Due to a severe skewness to this question (1.81), the variable was dichotomized as “*0* = <*4 days pr. week*” and “*1* = *More than 4 days pr. Week*.”

#### Dependent Variables

For those variables with a 7-point Likert scale, an eighth response option “*I don't know/not relevant*” was given. For all analyses, these participants were excluded. Table 2 presents the dependent variables in this study.

Participants' views of the use of salt on foot and cycle paths was measured using the statement “*Too much salt is used on foot and cycle paths*.” Answers were recorded using a 7-point Likert scale ranging from “*1–totally disagree*” to “*7–totally agree*.” The variable was dichotomized so that those who answered “*agree*” (alternative 5, 6 or 7) were coded as 1 and those who reported “*disagree*” (alternative 1, 2 or 3) or were neutral (4) were coded as 0. Only the 1,743 (73%) respondents who reported to at least sometimes cycle during winter were presented with this question.

Satisfaction with snow plowing on foot and cycle paths was measured asking “*How well do you think foot and cycle paths were maintained in your municipality last winter with regards to: snow plowing*?” Participants could answer using a 7-point Likert scale ranging from “*1–very poor*” to “*7–very good*.” The variable was dichotomized so that those who reported “*good*” (alternative 5, 6 or 7) were coded as 1 and those who reported “*poor*” (alternative 1, 2 or 3) or were neutral (4) were coded as 0. Only the 1,743 (73%) respondents who reported to cycle at least sometimes during winter were presented with this question.

Encountering shards of glass can be annoying, but also dangerous. How often cyclists experience this will affect how problematic it is to them. This question was phrased as “*How much of a problem are shards of glass in the road for you, when you cycle during summer.”* Participants could answer using a 7-point Likert scale ranging from “*1*–*no problem at all*” to “*7*–*very problematic*.” The variable was dichotomized so that those who ticked alternatives 5, 6 or 7 were coded as 1 and those who ticked alternatives 1–4 were coded as 0.

Potholes and uneven roads were asked about in a similar way “*How much of a problem are potholes and uneven roads for you, when you cycle during summer.”* Participants answered using a 7-point Likert scale ranging from “*1*–*no problem at all*” to “*7*–*very problematic*”.

The variable was dichotomized so that those who ticked alternatives 5, 6 or 7 were coded as 1 and those who ticked alternatives 1–4 were coded as 0.

Choosing not to cycle due to poor conditions was measured using the question “*Do you sometimes forfeit cycling during summer due to difficult conditions?”* Respondents answered either ≪*yes*≫ (1) or ≪*no*≫ (0).

Accidents where conditions contributed was only asked to those 711 (30%) respondents who reported having experienced a cycle accident. The question was phrased as “*Did you experience an accident where conditions played a part?*”

How often participants cycle during winter was asked “*How often do you cycle during winter?*” and had four alternatives “1–Never,” “2–Occasionally,” “3–Sometimes,” and “4–Often.” This was dichotomized as “*0* = *Never/occasionally/sometimes*” and “*1* = *Often*.”

### Analyses

All analyses were performed in SPSS 26. In the logistic regression models, a standardized effect size [OR/Exp(B)] is reported. Confidence intervals are omitted for brevity, and *p*-values are reported using the norm of *p* < *0.05* and the advised Bonferroni correction for multiple comparisons *p* < *0.008* (p/number of tests).

For all analyses, we report *p*-values. Results with *p*-values below 0.05 are regarded as statistically significant. Interpretations are additionally based on effect sizes (coefficients and differences). By focusing only on statistical significance, much information would get lost (Wasserstein, 2016; Amrhein et al., 2019).

For regression analyses, we included our measure of cycling frequency separately. By including this variable, we may control away some of the variation that we actually are interested in. However, when controlling for this, we get a better insight into participants' evaluation of the importance of maintenance and operations to cycling frequency. Therefore, models are calculated with multiple steps to investigate this effect.

## Results

### Bivariate Analyses

**Figure 2:**
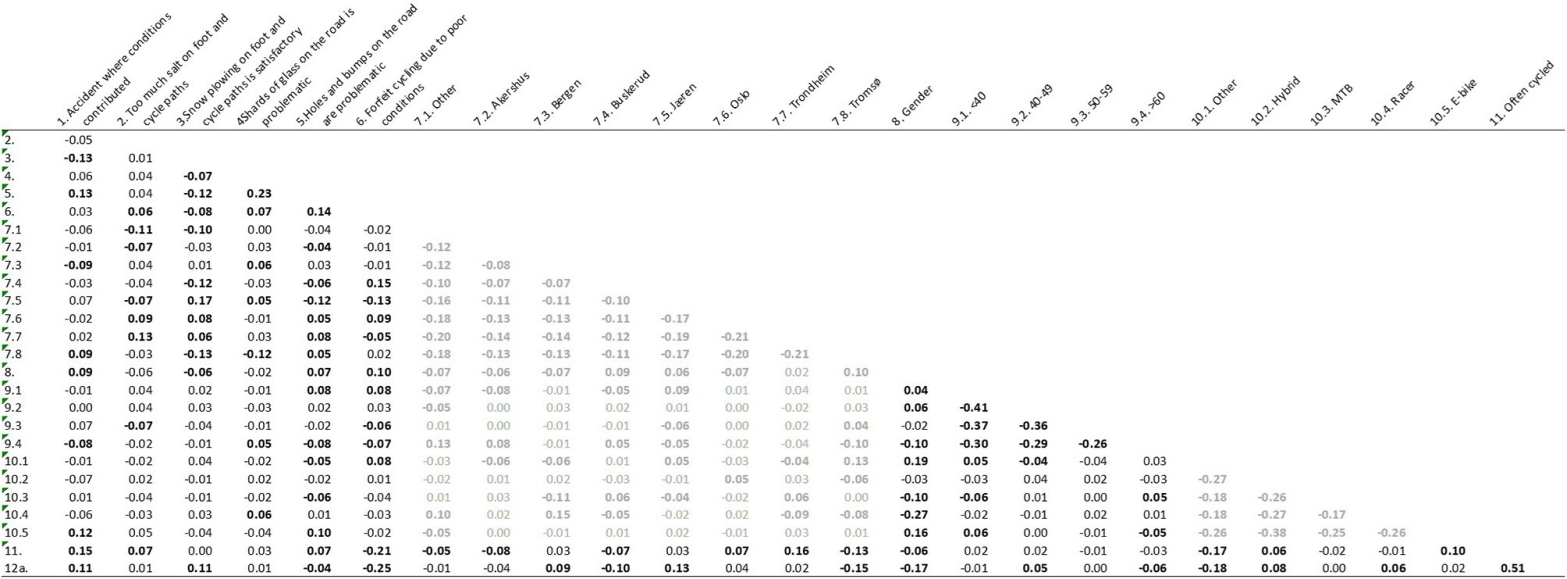
Bivariate correlations (Pearson's *r*) between all variables included in the regression models. Bold: *p* < 0.05. 12a = Winter cycling. *N* = 2,376. Coefficients of reduced interest are presented in gray for readability.

To allow for direct investigation of relationships of all included variables, we performed a bivariate correlation. This matrix is presented in Figure 2.

Several relationships between dependent variables emerge as statistically significant. For example, those who have had an accident where conditions contributed do more often think that current snow plowing is insufficient and are more likely to experience holes as problematic. Similarly, those who sometimes forfeit cycling due to poor conditions also experience the other variables as problematic.

Geographical areas also yield multiple significant coefficients. Respondents from Oslo and Trondheim are more likely to agree with the statement that maintainers use too much salt. Shards of glass are least problematic in Tromsø. Respondents from Buskerud and Oslo are more likely to forfeit cycling due to poor conditions.

In contrast to the elderly, young people are more likely to experience problematic holes and bumps in the road. Elderly also have fewer accidents where conditions contributed but are more bothered by shards of glass on the road. Females cycle less often than males.

Cyclists using racer bikes are more likely to experience shards of glass on the roads as problematic. E-bike users are more susceptible to potholes and to experience accidents where conditions contributed.

### Regression Models

**Figure 3:**
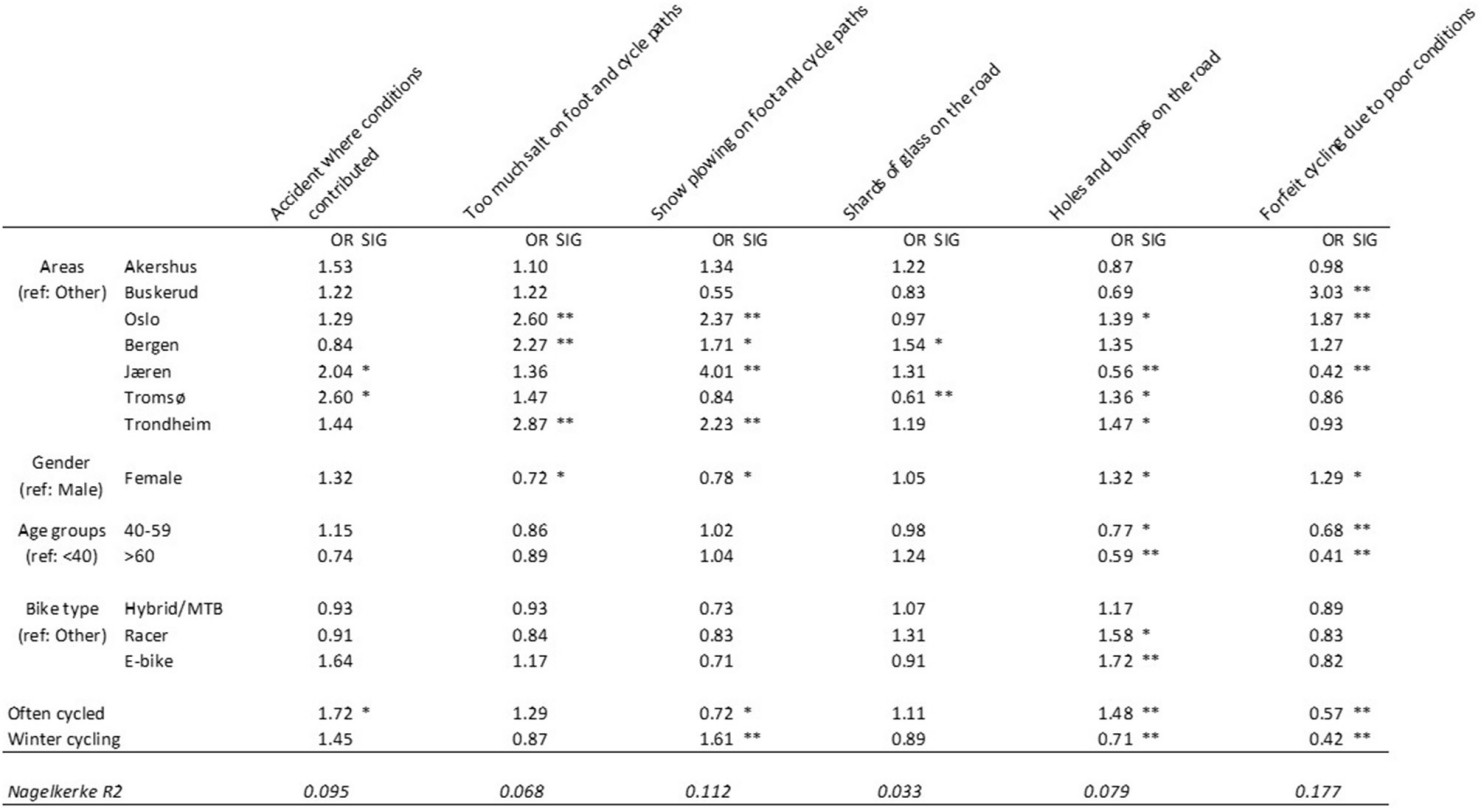
Six hierarchical logistic regression models predicting six different measures of impact suffered due to conditions or M&O. OR, Odds ratio [EXP(B)]. *p < 0.05, **Bonferroni corrected *p* < 0.008. *N* = 2,376.

When incorporating multiple predictor variables in the same model, one can investigate the effect of each variable while controlling for the effect of the others. Six separate hierarchical regression models are presented in Figure 3. Cycling frequency is included in step 2.

Few coefficients change a lot when cycling frequency is introduced. The effects of *E-bikes* and *Racer* seem to reliably diminish. Cycling frequency seems particularly important in predicting accidents where conditions contributed and forfeiting cycling due to poor conditions.

Those who cycle often report experiencing an accident where conditions contributed to a larger extent than those who cycle seldom. Respondents from *Jæren* and *Tromsø* experience such accidents about twice as often as cyclists from other regions. Only respondents from *Bergen* score lower than the *Other* reference category. E-bike riders also report experiencing such accidents more often than others, although this coefficient is not statistically significant when controlling for cycling frequency.

Females are less critical of the amount of salt used then men, and younger participants are least likely to think that too much salt is used. Those who live in *Trondheim, Oslo*, and *Bergen* are more critical to the use of salt than respondents from other areas.

In *Buskerud* and *Tromsø* respondents are less satisfied with snow plowing than in other regions. *Oslo, Bergen, Jæren* and *Trondheim* have citizens who are relatively more satisfied with snow plowing on foot and cycle paths. Males are more satisfied than females.

Cyclists in *Buskerud* and *Jæren* report less problems with holes and bumps in the road than cyclists from other regions. Males report this to be less of a problem than females, and older cyclists less than younger ones. Those who cycle during winter also are less bothered by this than those who don't. Participants from *Oslo, Tromsø, and Trondheim* report experiencing more problems with holes than those from *Other* areas. Racer-bikes, E-bikes, and those who generally cycle more often are also more susceptible to experiencing problematic holes. These bike types may often hold higher speeds than the rest, which may exacerbate the problem for them.

All bike types experience not executing a planned trip due to poor conditions less frequently than *Other* bike types. Here, it seems that the effect of bike type is to an extent impacted by differences in cycling frequency. Participants in *Buskerud* and *Oslo*, as well as females, also report forfeiting cycling more often than *Other* areas and men. This happens less frequently to participants in *Jæren*, older participants and those who cycle more in general. Not surprising, cycling frequency is positively associated with having experienced an accident where conditions contributed, and it is negatively associated with forfeiting to cycle due to poor conditions.

#### Winter Cycling

Cycling conditions change a lot through the year in countries with snowy winters. Investigating which factors are important in determining whether one cycles during winter, may yield additional information about the importance of M&O. Whether one chooses to cycle during winter or not could be an important proxy for their evaluation of conditions for winter cycling. Table 3 presents a hierarchical logistic regression that predicts winter cycling.

**Table 3:** Hierarchical logistic regression model predicting winter cycling in three steps.

		**Step 1**		**Step 2**		**Step 3**	
		**OR**	**SIG**	**OR**	**SIG**	**OR**	**SIG**
Areas (ref: Other)	Akershus	0.91		0.90		0.83	
	Bergen	1.65		1.70		1.60	
	Buskerud	0.61		0.46	^*^	0.49	^*^
	Jæren	1.88		1.75	^*^	1.49	
	Oslo	1.64		1.24		1.12	
	Trondheim	1.56		1.02		0.96	
	Tromsø	0.78		0.72		0.73	
Gender (ref: Male)	Female	0.72	^*^	0.71	^*^	0.71	^*^
Age groups (ref: <40)	40–49	1.34		1.32		1.30	
	50–59	1.20		1.24		1.25	
	>60	0.98		0.93		0.93	
Bike type (ref: Other)	Hybrid	1.87	^*^	1.50		1.55	
	MTB	1.40		1.41		1.43	
	Racer	1.57		1.36		1.37	
	E-bike	1.58	^*^	1.14		1.18	
Often cycled		–		9.135	^**^	9.60	^**^
Too much salt	–		–			0.84	
Satisfied with snow plowing	–		–			1.65	^**^
Nagelkerke *R*^2^	0.061		0.288			0.300	

*OR, Odds ratio [EXP(B)]*.

* *p < 0.05*,

** *p < 0.001*.

It seems that cyclists in Buskerud experience M&O as quite poor, as they cycle markedly less during winter (see also Figure 3). In Jæren, with its mild climate, cycling is less dependent on seasonal variations. Gender also seems to play a role, and females cycle less during winter then men. The effects of bike type lessen as cycling frequency is controlled for. It is also important to remember that bike type was reported during summer, and many may use a different bike during winter. There is also a non-significant trend that the middle-aged participants cycle relatively more during winter. Those who are more satisfied with snow plowing also seem to cycle more during winter, even when controlling for other factors.

## Discussion

There are many factors to consider when authorities want to increase the use of bicycles for transport. In this paper we have investigated some factors related to maintenance and operation, that were highlighted by previous research. Some groups and demographics suffer more from poor conditions than others, and geographic variation is important to consider. For most problems women are more vulnerable than men. However, according to our survey, elderly cyclists are not particularly affected by poor conditions, the did for instance not consider holes in the surface to be such a great problem. This section will discuss the proposed hypotheses and the exploration of data. Limitations are considered.

### Female Cyclists Will Be More Affected by Poor Cycling Conditions Than Men

Previous research has found that female cyclists are particularly prone to suffer from poor conditions and M&O (Svorstøl et al., 2017; Johansson and Bjørnskau, 2020). While the mechanisms through which these effects occur remain unexamined, we confirm our hypothesis to some extent in this study. Multiple regression models suggest that women more often forfeit cycling due to poor conditions, suffer more from holes, and more often than men have experienced accidents where conditions contributed. Women also cycle less than men during winter when controlling for cycling generally, which may be a sign that they are more impeded by difficult conditions.

Bivariate results show multiple significant relations. Women report cycling less than men, which may influence their experience with different kinds of conditions. It may also be that they find current road conditions too poor to increase their cycling. This finding is also in line with previous research finding that females perceive cycling as less available to them (Sahlqvist and Heesch, 2012).

As previous research has noted, the gender differences are more visible when conditions are particularly bad, such as icy roads and heavy snow (Johansson and Bjørnskau, 2020). This could mean that the effects found in this study will be even further accentuated when asking about more adverse conditions. It is nonetheless a problem that needs further scrutiny to be fully understood.

These results indicate that difficult conditions are a more pressing issue for female cyclists. The way current M&O is executed may thus be an important barrier for more women to start cycling. Previous research in a Norwegian context has suggested that women are more cautious in traffic than men (Bjørnskau, 2005). It is reasonable to assume that such cautiousness leads to women regarding difficult conditions as riskier than do men. A recent study also indicated that females have higher risk when riding e-bikes compared to conventional bikes, and compared to the risk for men on e-bikes (Fyhri et al., 2019). These factors could help explain the difference we find in our study, which highlights the need for further investigation of differential effects of adverse conditions for cyclists.

### Older Participants Will Be More Affected by Poor Cycling Conditions Than Younger Participants

Our second hypothesis stated that older participants would be more affected by adverse conditions than younger ones. The results from the current study do not support this hypothesis. In the regression models, there are only statistically significant results of age when assessing whether holes pose problems and whether they sometimes forfeit cycling. Both are also opposite of what one would expect given our hypothesis; older people find holes less problematic and are less prone to forfeit cycling due to poor conditions. This is true regardless of the control for cycling frequency, and seems contrary to other findings that younger cyclists are prone to accidents and risky behavior (i.e., Useche et al., 2019; Bjørnskau, 2020). However, many factors other than age influences how affected a bicyclist is by poor cycling conditions. Some were not included in this study, such as cycling intensity, weather avoidance, or self-reported risky behavior. Future research should further investigate the importance and interactions with such human factor measures.

The correlation matrix suggests that several relationships for the oldest participants are larger than what we would expect if the null hypothesis was true. They are less likely to have experienced an accident where conditions contributed, less bothered by holes and bumps, and less likely to forfeit cycling. This also strengthens our conviction to reject our hypothesis, as the effects are robust regardless of control for cycling frequency–these results are not due to other factors such as elders generally cycling less than the general populace.

While beyond the scope of this article, other research suggests an interaction effect between age and gender, especially for some kinds of conditions (Johansson and Bjørnskau, 2020), indicating that elderly women are the ones most affected by poor cycling conditions. This could also particularly be true for adverse conditions such as ice or heavy snow. Our data is also insufficient to further explore the somewhat surprising find that participants under 40 are more bothered by some poor conditions. One explanation could be that younger cyclists more often cycle with children, either in a separate trolley or in a children's seat. Another possibility is that elderly may cycle more for recreational purposes while the younger cyclists more often cycle for transport. There could also be a self-selection bias that is more accentuated among elders, that those who keep cycling are the ones who find conditions the least problematic. It is important to remember that oldest age group in this study was defined as 60 years+. Many of these cyclists are not very old and probably very experienced and quite fit, and possibly those most inclined to participate in a survey about cycling. This could create a difference in assessments of conditions such as the ones in the current article. Additionally, children have been found to have delayed reaction times and inefficient visual search patterns in traffic situations (Zeuwts et al., 2017), further emphasizing the different issues associated with different age groups.

### Maintenance and Operation Affect Whether One Chooses to Cycle

Particularly two of the dependent variables in this study are key to investigate this hypothesis. About three fourths of our participants have experienced at least one accident where conditions contributed. Experiencing such an accident could inhibit people to cycle, and one third sometimes forfeit cycling due to poor conditions. Therefore, we conclude that our hypothesis is confirmed. These kinds of results have been expanded upon by previous research (Johansson and Bjørnskau, 2020). Here we have confirmed this effect and explored whether such effects were different for different groups. While we argue that the current study builds the case that M&O affects whether one chooses to cycle, more research should investigate this effect and how it impacts the perception of arduous cycling conditions.

### Geographic Variations

Our selection of geographic areas covers quite large differences in climate. This is also evident when considering results and consistent with previously reported survey results (Johansson and Bjørnskau, 2020). These results did, however, not include multivariate analyses. Inhabitants in all included areas are more critical of the use of salt than the *Other* areas, particularly in Oslo, Bergen and Trondheim. This is a debated topic because while salt is very effective in removing ice and keeping roads and pavements bare, it wears down the bicycle and has negative effects on the environment. Our results suggest that people in city areas are more critical of salt-use than those from more rural areas. Inhabitants in *Jæren* seem very pleased with current levels of snow plowing, but this could be because of their limited exposure to snow. They are, also least bothered by problematic holes, which may indicate more satisfaction with M&O in general as they more seldom forfeit cycling due to poor conditions.

Participants from *Buskerud* and *Oslo* are most likely to forfeit cycling due to poor conditions. Cyclists from *Buskerud* do not produce statistically significant coefficients in other variables, which may indicate that different conditions than those included are problematic. This is also evident in regression models predicting winter cycling, where they are the least likely to cycle during winter. For cyclists in *Oslo*, too much salt and holes in the road may be to blame. While they are more satisfied with snow plowing and generally cycle more during winter.

Based on regression models, there is no reason to conclude that some areas are definitively worse than others. It seems they all have their issues and unique challenges, as their answers vary. However, forfeiting to cycle due to poor conditions, even while controlling for other variables, is a serious result. Based on this variable, it seems that *Jæren* may has a generally milder climate and thus suffer less from seasonally induced adverse conditions and wear on the asphalt. This should mean that other similar areas such as *Bergen* be rated similarly, which it is not. This may indicate that cycling infrastructure and M&O is better in Jæren than in Bergen. These results highlight the importance of control for geographic variations in future studies.

### Limitations

The study is based on a self-report questionnaire administered at one point in time. This means that results are affected by any bias that results from self-reporting one's own behavior and cognitions. This also means that respondents had to rely on memory when assessing how conditions were last winter, which for some was half a year in the past. The study sample may also include some self-selection bias; how much one chooses to cycle or whether one chooses to cycle during winter could be an important proxy for their evaluation of cycling conditions.

It is important to remember that the survey was administered during summer, so bike type may not be as relevant for winter conditions. *Racers* may be used less during winter, which may in turn impact which conditions cyclists find difficult. This could hide some effects of bike type during winter.

As Norwegians are allowed to cycle on pavements, formulations about road surface may not be unambiguous. There are differences in who chooses to cycle in car lanes and who chooses to cycle on pavements that may impact the results reported in this study. The survey administered in this study was at times aimed at investigating the surface condition of whatever surface the responding cyclist used to bike on, while sometimes clearly referring to specific kinds of infrastructure. We recommend that future research seeks to investigate these factors further. Additionally, there may be differences between cities in how much and what kind of cycling-specific infrastructure is available, which may exacerbate differences between them.

Another limitation is our limited control for cycling frequency (“*How often do you normally cycle this time of year”*). While only truly crucial for measures of absolute outcomes, a crude proxy such as ours does limit our ability to conclude about accident risk. It is to be assumed that those with racer bikes cycle more and longer than the rest, which may in turn impact a range of included variables. The collinearity of variables in regression models could also complicate results, although no extreme correlation was uncovered during analyses (cf. Figure 2). The measures included were always meant to be treated as relative, not absolute. When considering the explained variance in each model (Nagelkerke *R*^2^), we see that there is much variation that is left unexplained. Particularly whether one perceived shards of glass on the road as problematic is poorly explained. For forfeiting cycling due to poor conditions, the explained variance (Nagelkerke *R*^2^) is considerably higher. Forfeiting to cycle due to poor conditions is a ≪broad≫ issue and something most cyclist can relate to and have considered. Shards of glass is a much more specific problem, probably only experienced by some cyclists. Hence, it is not surprising that explained variance differ between the two models.

While generally not advised, it can sometimes be the best solution to dichotomize variables (MacCallum et al., 2002). This limits the information and variation in the data set, which weakens the ability to draw inferences. However, because most variables in this sample were highly skewed, we decided to dichotomize.

### Implications

These findings have clear implications for future M&O. In their pursuit to incentivize more cycling, authorities should prioritize certain areas more than others. As it is today, Norwegian cyclists are most bothered by holes and bumps in the road, shards of glass and insufficient snow plowing. However, not all groups of cyclists are equally affected by this, and geographic variations are important. Surprisingly, it seems that older cyclists are less bothered than younger ones by some difficult conditions. They are also less likely to forfeit cycling. There might be a self-selection bias explaining this—only those who still cycle have been included in the analysis, and the elderly participants may be particularly experienced and fit compared to the average elderly population. It is indeed possible that many elderly, who do not cycle and hence are not included in this sample, could potentially have cycled if the conditions were better. This effect could also extend to other parts of the results, as participants were quite experienced and chose to partake in the study voluntarily. Thus, it is difficult to conclude what measures the authorities should use to get more people to start cycling, but what we clearly see is that better maintenance and operations could in general increase cycling use, and in particular for women and for cyclists under 40. This could be on typical commuter stretches or close to kindergartens.

Given that female cyclists are more troubled by poor conditions and more risk aversive than men, improved maintenance and operation is particularly important if one wants to increase cycling among women.

## Conclusions

Norwegian authorities want to increase the share of transportation done by cycling, particularly in city areas. To achieve this, and to do so safely, more information about maintenance and operation and how cyclists perceive conditions is needed. This article reports a nation-wide survey to 2,376 cyclists across Norway. Our results suggest that females suffer more from adverse conditions than do males. Furthermore, we find that females are less likely to cycle during winter, which may be an important proxy for their perception of winter conditions for cyclists. In contrast, older cyclists in our sample do not seem to be as affected as younger cyclists. Cycle type also matters; cyclists on racers and e-bikes are more bothered by holes in the road than riders of other cycle types. Geographic variation has a great impact on the effects of cycling conditions, and it is also evident that the cyclists' perceived quality of maintenance and operation varies greatly between geographical areas. Improved maintenance and operation could promote cycling especially for women and the potential seems to be much larger in some areas than others.

## Data Availability Statement

The datasets presented in this article are not readily available because they are anonymized. Requests to access the datasets should be directed to the corresponding author.

## Ethics Statement

The studies involving human participants were reviewed and approved by NSD–Norwegian Centre for research data. The patients/participants provided their written informed consent to participate in this study.

## Author Contributions

OA was chiefly responsible for the literature review, analysis, and text production. TB was chiefly responsible for the discussion chapter and general tips and revision. All authors contributed to all parts of this study.

## Conflict of Interest

The authors declare that the research was conducted in the absence of any commercial or financial relationships that could be construed as a potential conflict of interest.
